# Prostatic relapse of an undifferentiated teratoma 24 years after orchidectomy

**DOI:** 10.1186/s13104-015-1445-9

**Published:** 2015-10-01

**Authors:** Tobias Janowitz, Sarah Welsh, Anne Y. Warren, Jane Robson, Benjamin Thomas, Ashley Shaw, Nicola L. Ainsworth, David E. Neal, Danish Mazhar

**Affiliations:** Oncology Department, Addenbrookes Hospital, Hills Road, Box193, Cambridge, CB2 OQQ UK; Cancer Research UK Cambridge Institute, University of Cambridge, Li Ka Shing Centre, Robinson Way, Cambridge, CB2 0RE UK

**Keywords:** Germ cell tumour, Stage I teratoma, Late relapse, Prostatic metastasis, Tumour markers

## Abstract

**Background:**

Non-seminomatous germ cell tumours make up about 40 % of all germ cell tumours, which in turn are the most common tumours in men aged 15–44 years. Low risk stage I non-seminomatous germ cell tumours, which are confined to the testes, are commonly treated by orchiectomy and surveillance. Up to 20 % of patients with this diagnosis relapse, usually within 1–2 years of follow up, but very rarely after more than 5 years. The most common sites of relapse are the retroperitoneal lymph nodes, the mediastinum, and the lungs. We describe a case of relapse in the prostate over 20 years after initial diagnosis, which has not been described in the literature so far.

**Case presentation:**

This report presents a 49-year-old white British man with relapsed testicular non-seminomatous germ cell tumour 22 years after initial treatment with orchidectomy only. He relapsed with a prostatic mass, haematospermia and back pain. His prostate specific antigen levels were within normal range. Alpha feto-protein and lactate dehydrogenase levels were elevated, and his human chorionic gonadotrophin levels were normal. A biopsy confirmed undifferentiated malignant tumour, shown immunohistochemically to be a yolk sac tumour. The patient was initially treated with bleomycin, etoposide and cisplatin chemotherapy, but developed bleomycin-related pulmonary side effects after two cycles. His treatment was changed and he completed four cycles of chemotherapy by receiving two cycles of etoposide, ifosfamide, and cisplatin. Post treatment blood tumour markers were normal, but a follow up computed tomography showed a mass in the base of the prostate, the trigone and the left distal ureter which was surgically resected. The histology from the surgical resection was of necrotic tissue. The patient is now in follow up at 3 years after treatment with no evidence of residual disease on computed tomography. His Alpha feto-protein, beta human chorionic gonadotrophin and lactate dehydrogenase levels are normal.

**Conclusions:**

Very late relapse in stage I non-seminomatous germ cell tumours is extremely rare and the prostate is a highly unusual site of relapsed disease. For diagnosis of late relapse, this case confirms the value of serum biomarkers in germ cell tumours, in particular non-seminomatous germ cell tumours.

## Background

Non-seminomatous germ cell tumours (NSGCTs) constitute about 40 % of all germ cell tumours, which in turn are the most common tumours in men aged 15–44 years [1]. After orchidectomy, the risk of relapse in stage I disease can be predicted by histological examination of the orchidectomy specimen for the presence of lymphovascular invasion. If this is absent the relapse risk is 20 %; if it is present the risk is 50 % [2–4]. This risk is accumulated mainly in the first 2 years. Later relapse is extremely rare [5–7]. The retroperitoneum is the most common site of relapse, followed by mediastinal, lung and pleural, and rarely pelvic lymph nodes [8]. In the case of relapse, the cure rates are 98–99 % due to the high sensitivity of NSGCTs to platinum based chemotherapy. Adjuvant retroperitoneal lymph node dissection and adjuvant chemotherapy have been shown to reduce relapse risk, but have side effects and cost implications, and are considered mainly in high-risk patients [9]. Post treatment surveillance comprises of history, examination, measurement of the tumour markers beta human chorionic gonadotrophin (beta HCG) and alpha feto-protein (AFP), and radiological surveillance with chest radiographs and computed tomography (CT) at regular intervals. Radiological investigations and measurements of tumour markers, in cases with initially raised marker levels, are the most important parts of the surveillance protocol [10]. There is no consensus regarding the number or frequency of CT scans on surveillance though results from the TE08 trial suggest 2 CT scans of the abdomen at 3 and 12 months of follow-up may be sufficient [11, 12].

In contrast to the large majority of cases, we present a case of relapsed stage I NSGCT more than 20 years after orchidectomy without adjuvant therapy. This case is further noteworthy due to the highly unusual site of relapse within the prostate.

## Case presentation

**Fig. 1:**
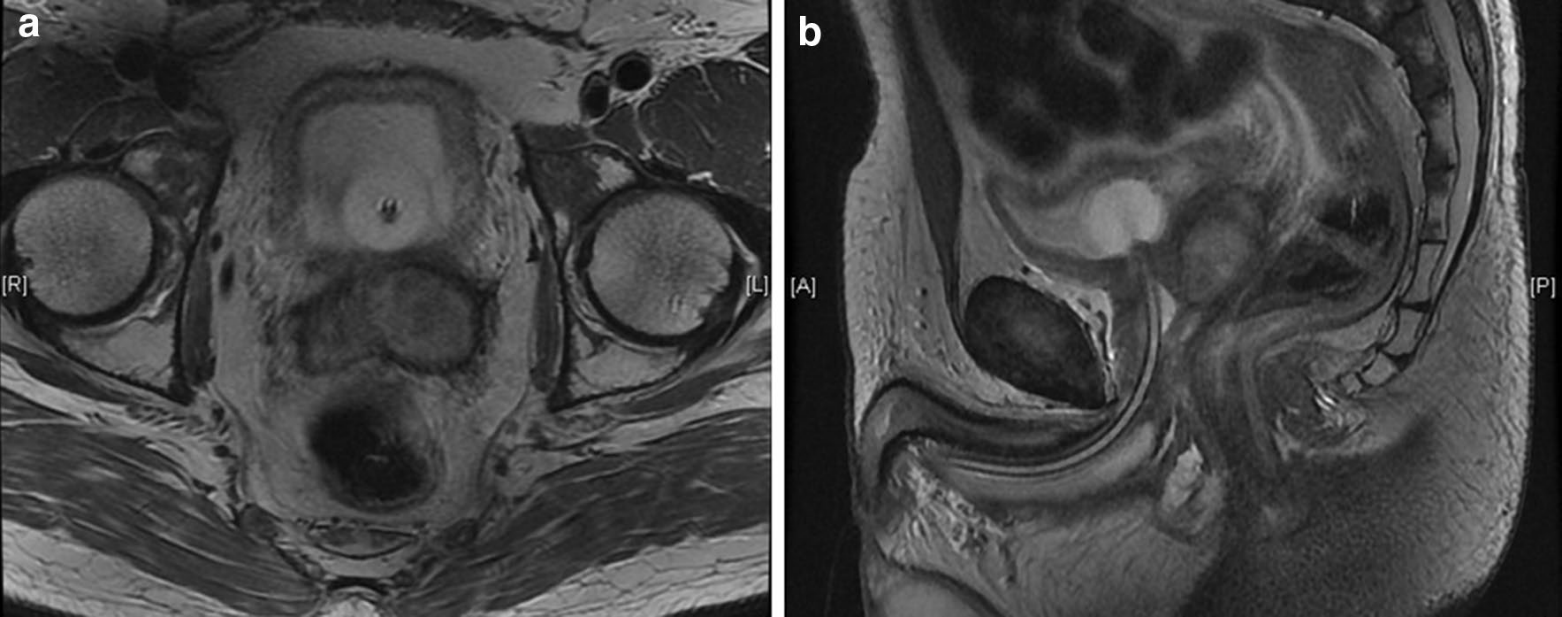
T2-weighted magnetic resonance images. **a**, **b** Axial and coronal T2-weighted magnetic resonance images demonstrate a heterogeneous high signal lesion in the left seminal vesicle and prostate gland. There is a urinary catheter in situ. The lesion is markedly different to the low signal lesion typically found in primary prostatic carcinoma

**Fig. 2:**
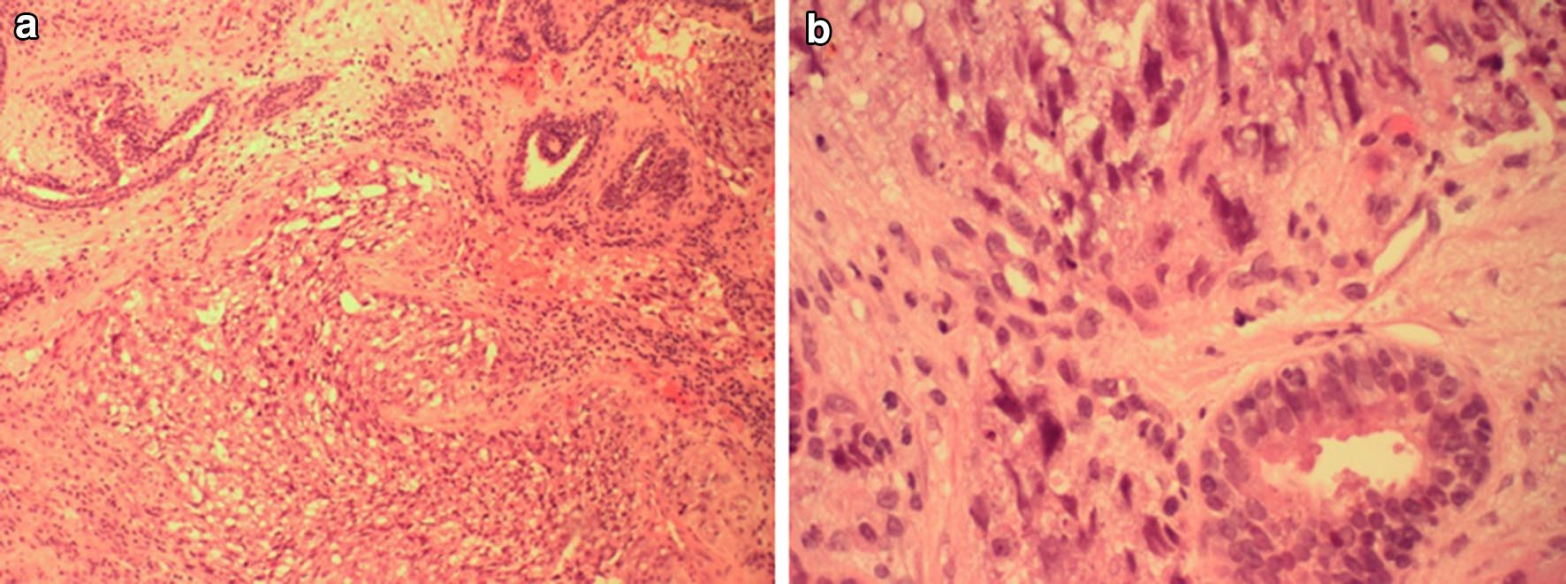
Histopathological characterisation. Analysis was performed on paraffin-embedded tissue using haematoxylin and eosin (HE) stained sections. **a** ×10 magnification showing benign prostatic glands (*upper part* of picture) and undifferentiated tumour with sheets of cells and small cystic spaces (*lower part* of picture). **b** ×40 magnification showing a normal benign prostatic gland (*lower part* of picture) and undifferentiated tumour (*upper part* of picture)

A 49-year-old white British man presented to his General Practitioner and afterwards a Urologist with spontaneously resolving haematospermia, normal prostate specific antigen (PSA) and normal magnetic resonance imaging (MRI) of the prostate. His past medical history was remarkable for stage I NSGCT which was treated with orchidectomy 21 years previously. He took no regular medication. A year after resolution of the haematospermia he developed nocturia, poor urinary flow, and a feeling of pressure on his rectum. This was associated with severe back pain, for which he had started simple analgesia. Examination and ultrasound investigation of the remaining testis was normal. His PSA level was in the normal range at 1.11 microg/L but his AFP level at that stage was grossly elevated at 7787 ng/mL (normal range <10 ng/mL) with an lactate dehydrogenase (LDH) level of 486 U/L (normal range 0–250 U/L). HCG and free beta HCG levels were normal at less than 2 and less than 0.2 U/L, respectively. CT at that point demonstrated a pelvic mass with associated enlarged pelvic lymph nodes, mild left hydronephrosis, and multiple pulmonary metastases up to a diameter of 1.7 cm. An MRI was subsequently performed to better define the local anatomy prior to surgery. MRI demonstrated an extensive prostatic tumour extending into bladder (Fig. 1a, b), seminal vesicles, rectum and left ureter causing hydronephrosis. The total volume of the prostate was elevated at 113 mL. Histopathology from bladder biopsy confirmed the diagnosis of an undifferentiated malignant tumour that from its immunoprofile (AFP, PLAP and CD117 positive, CD30 and OCT 3/4 negative), was diagnosed as metastatic yolk sac tumour (Fig. 2). Due to the non-pulmonary visceral metastases in the prostate his disease fell into the high risk category and treatment with four cycles of bleomycin, etoposide, cisplatin (BEP) chemotherapy was initiated. He received a nephrostomy for decompression of the hydronephrosis and completed the first two cycles of BEP chemotherapy, but then developed evidence of bleomycin-related lung changes on chest radiography and CT. His treatment was changed to etoposide, ifosfamide, and cisplatin (VIP) chemotherapy and he completed another two cycles for a total of four cycles of chemotherapy. He did not have any long term effects from the bleomycin.

All tumour markers were within the normal range after completion of chemotherapy. A CT 3 months into follow up, however, showed a residual mass of 2.7 cm in the base of the prostate, the trigone and the left distal ureter which was surgically resected. The excision specimen showed necrotic tissue and small amounts of normal prostate tissue and fibrosis, but no evidence of tumour cells. The tumour markers at this point had completely normalised. The patient is now in follow up at 36 months after treatment with no evidence of residual disease on CT. His AFP, beta HCG and LDH levels are normal.

## Conclusion

The incidence of relapse in stage I NSGCT later than 2 years after surgery is very low. No large data sets are available on late relapse, but published case series suggest, that the majority of relapses in non-seminoma patients occur in the first 10 years of follow up, with the retroperitoneum being the most common site of relapse, followed by the mediastinum, the lung and pleura, and rarely the pelvic lymph nodes [5, 6, 8, 13–16]. There are a few cases published for relapse of germ cell tumors >20 years after diagnosis [17, 18]. Reviewing the pathology of relapses later than 2 years, Michael et al. found teratoma was the most commonly identified histology followed by yolk sac. Unusual types of yolk sac tumor were found to cause differential diagnostic problems with non-germ cell carcinomas, especially as there were many different sites of relapse [14].

Two case reports are similar to this case, Arafat et al. describe a case involving a patient with stage I NSGCT who relapsed with a retroperitoneal mass after 27 years [19] and Lattouf et al. a case of stage I NSCGT who presented with a seminal vesicle relapse after 20 years [20]. For seminoma combined with yolk-sac tumour, late relapse after 43 years of surveillance has been described [21]. Notably, the published literature on case series in seminoma suggests that late relapse beyond 10 years is more common than for non-seminomas [7, 8, 22].

The patient described in this report had relapsed disease after 22 years from a stage I NSGCT that had been treated with orchidectomy and surveillance. Therefore, this case is distinct from the majority of reports listed above due to its stage, protracted time to relapse and site of relapse.

The tumour marker alpha fetoprotein was grossly elevated at relapse and the tumour was visible on radiological investigation. Current guidelines do not cover in detail the protocol for continuous follow up of patients with stage I non-seminoma due to the lack of quality evidence [23, 24]. Surveillance is more rigorous at the start as most relapses are early-median time to relapse for NSGCT is around 6–9 months. This includes monitoring of the tumour markers and radiological investigations [23, 24].

One case report is, of course, insufficient to inform the treatment and follow up guidelines. It does, however, confirm the value of tumour marker in diagnosis of very late relapsed non-seminoma. Furthermore, it establishes evidence that NSGCT can recur more than 20 years after initial treatment, and this can be in highly unusual sites such as the prostate. It also highlights the need to note the history of a previous germ cell tumour when late metastatic disease occurs, as the histological features of such tumours (particularly yolk sac tumours post chemotherapy) may not be typical of the primary tumour and may therefore be misinterpreted histologically.

 Written informed consent was obtained from the patient for publication of this case report and any accompanying images.

## Abbreviations

NSGCT: non-seminomatous germ cell tumour
PSA: prostate specific antigen
AFP: alpha feto-protein
LDH: lactate dehydrogenase
HCG: human chorionic gonadotrophin
CT: computed tomography
BEP: bleomycin, etoposide and cisplatin chemotherapy
VIP: etoposide, ifosfamide, and cisplatin chemotherapy
MRI: magnetic resonance imaging
